# Molecular and Electrophysiological Characterization of GFP-Expressing CA1 Interneurons in GAD65-GFP Mice

**DOI:** 10.1371/journal.pone.0015915

**Published:** 2010-12-31

**Authors:** Corette J. Wierenga, Fiona E. Müllner, Ilka Rinke, Tara Keck, Valentin Stein, Tobias Bonhoeffer

**Affiliations:** 1 Department of Cellular and Systems Neurobiolgy, Max Planck Institute of Neurobiology, Martinsried, Germany; 2 Synaptic Receptor Trafficking, Max Planck Institute of Neurobiology, Martinsried, Germany; Tokyo Medical and Dental University, Japan

## Abstract

The use of transgenic mice in which subtypes of neurons are labeled with a fluorescent protein has greatly facilitated modern neuroscience research. GAD65-GFP mice, which have GABAergic interneurons labeled with GFP, are widely used in many research laboratories, although the properties of the labeled cells have not been studied in detail. Here we investigate these cells in the hippocampal area CA1 and show that they constitute ∼20% of interneurons in this area. The majority of them expresses either reelin (70±2%) or vasoactive intestinal peptide (VIP; 15±2%), while expression of parvalbumin and somatostatin is virtually absent. This strongly suggests they originate from the caudal, and not the medial, ganglionic eminence. GFP-labeled interneurons can be subdivided according to the (partially overlapping) expression of neuropeptide Y (42±3%), cholecystokinin (25±3%), calbindin (20±2%) or calretinin (20±2%). Most of these subtypes (with the exception of calretinin-expressing interneurons) target the dendrites of CA1 pyramidal cells. GFP-labeled interneurons mostly show delayed onset of firing around threshold, and regular firing with moderate frequency adaptation at more depolarized potentials.

## Introduction

GABAergic interneurons comprise ∼10–20% of the total neuronal population and are essential for controlling and synchronizing the output of the principal cells [1]–[3]. There are many different types of interneurons, executing diverse functions in shaping the activity of neuronal networks. It has proven difficult to formulate an unequivocal definition of the different interneuron types that exist in the brain [4]. Recent work describing the origin and development of different interneuron types has contributed greatly towards solving this issue. Cortical and hippocampal interneurons were shown to be born outside of the cortex in the ventral telencephalon and to migrate tangentially during development to their final location in the adult brain [1], [5]. The majority of GABAergic interneurons originate from the medial ganglionic eminence (MGE) or the caudal ganglionic eminence (CGE) [6]–[8]. In addition, a small fraction of interneurons are generated in the preoptic area [9], [10]. Interneurons with different origin form separate interneuron classes and display distinct cellular properties. A full understanding of the developmental relationship between different types of interneurons will greatly contribute to define an unambiguous interneuron classification.

In order to better understand the function of different types of interneurons, different lines of transgenic mice have been created in which specific subsets of GABAergic interneurons are labeled. GAD65-GFP mice [11] are being used in numerous studies by many different labs [12]–[15]. A subset of GABAergic cells in these mice is brightly labeled with GFP. These interneurons are found in most brain areas and the spinal cord [11]. GFP is already expressed during embryonic development, which makes these transgenic mice very suitable for developmental studies. In this study, we provide a detailed analysis of the molecular and electrophysiological profile of GFP-labeled cells in the hippocampal CA1 area of GAD65-GFP mice. We report that GFP-labeled cells are characterized by a high coincidence of reelin expression (suggesting they emanate from the CGE), axons targeting the dendritic layers, and regular firing properties.

## Methods

All experimental procedures were carried out in compliance with the institutional guidelines of the Max Planck Society and the local government (Regierung von Oberbayern; Statement of Compliance #A5132-01). All animals are sacrificed prior to the removal of organs in accordance with the European Commission Recommendations for the euthanasia of experimental animals (Part1 and Part 2). Breeding and housing as well as the euthanasia of the animal are fully compliant with the German and European applicable laws and regulations concerning care and use of laboratory animals.

### Immunohistochemistry

Adult GAD65-GFP mice (P50-100) were anesthetized with Ketamine (0.21 mg/g) and Xylazine (0.015 mg/g) and perfused transcardially with 0.1 M phosphate-buffered saline (PBS, pH 7.3–7.4), followed by 4% paraformaldehyde in PBS. The brain was removed from the skull, postfixed overnight in the same fixative at 4°C, and then transferred to 30% sucrose in PBS for at least 2 days. Coronal sections were cut on a freezing microtome at 30 µm thickness. Free-floating sections were rinsed 3–5 times with PBS with 0.1% Triton X, incubated in a blocker solution containing 0.4% Triton X-100 and 10% goat serum for 2 hours at room temperature. Primary antibodies were applied overnight at 4°C in 0.1 M phosphate buffer with 0.4% Triton and 5% goat serum. Following extensive washing, appropriate secondary antibodies were applied at a concentration of 1∶200.

The following primary antibodies were used in this study: chicken anti-GFP (Chemicon #06-896; 1∶1000), rabbit anti-GABA (Sigma A2052; 1∶2000), mouse anti-GAD67 (Chemicon MAB5406; 1∶2000), mouse anti-reelin (MBL CR50; 1∶500), rabbit anti-VIP (Immunostar #20077; 1∶500), mouse anti-parvalbumin (Swant PV235, 1∶2000), rat anti-somatostatin (Chemicon MAB354; 1∶500), rabbit anti-calretinin (Swant #7699/3H; 1∶1000), rabbit anti-NPY (Immunostar #22940; 1∶1000), mouse anti-CCK (Dr. Ohning, UCLA Cure #9303; 1∶1000), rabbit anti-calbindin (Swant CB-38a; 1∶5000). Secondary antibodies were conjugated with Alexa488, Alexa633 and Cy3 (Molecular Probes, Invitrogen).

Image stacks (375×375 µm, 512×512 pixels; Δz  = 1.5 µm) were acquired over the entire depth of the section with a Leica Confocal microscope (LCS SP2) and analyzed with ImageJ. For each coronal brain section, 2-4 images were taken from the CA1 area in each hippocampus. The imaged regions were chosen to maximize the number of GFP cells they contained. Expression of labeled proteins was determined by visual inspection of the raw or, in a few cases, Gaussian filtered (σ = 2 pixels) image stacks and was performed for each label independently before colocalization was addressed. Cells were considered positive if the somatic fluorescence was clearly (>∼15%) above the local background level in multiple depth levels. For antibodies that do not stain the nucleus (GAD67, somatostatin, NPY and reelin), brightly labeled cells with nuclear staining were considered false positive and discarded. The percentage of positive cells was determined per section and values are given as averages ± SEM over all sections.

Most antibodies resulted in clear staining of only a few positive cells without background staining, clearly distinguishing positive and negative cells. GABA immunostaining showed high extracellular background staining at the top and bottom of our sections. We therefore only analyzed the middle portion of them, where positive cells could be clearly distinguished from background. As the NPY antibody showed relatively high background staining in all cells, cells were only considered NPY-positive when they were clearly brighter than their neighboring cells. For some NPY-sections we blindly repeated the analysis to ensure reproducibility of our results. The CCK antibody also strongly stained blood vessels. Immunostaining with the reelin antibody resulted in intense labeling of some cells, while other cells only had a dim punctate labeling around their cell bodies. Only the cells with intense somatic labeling were considered reelin-positive. It was recently described that the punctuate labeling reflects binding of extracellular reelin by reelin-negative cells [16].

### Firing patterns

Transverse hippocampal slices were prepared from juvenile GAD65-GFP mice (P14–21) as described previously [17]. Whole-cell patch clamp recordings were made at room temperature using a Multiclamp 700B amplifier (Molecular Devices). Signals were digitized at 5 kHz (Digidata 1440A and pClamp 10.2 Software; Molecular Devices) and Bessel filtered at 2 kHz. Pipette solutions contained the following (in mM): 150 K-methyl sulfate, 4 KCl, 4 NaCl, 4 MgATP, 0.4 Mg GTP, and 10 HEPES and 30 µM Alexa Fluor 594. Recordings were not corrected for a liquid junction potential (predicted value: −14 mV). Firing patterns were recorded in current clamp by injecting 1-second current pulses of variable amplitude (ΔI  = 1–5 pA around firing threshold; ΔI  = 50 or 100 pA otherwise).

Resting membrane potentials (< −40 mV) were measured at zero holding current soon after gaining whole cell access. Series resistances ranged from 5 to 25 MOhm. Bridge-balance compensation was applied in a subset of experiments. Input resistances were measured by the steady state response to a 100 ms pulse of −5 mV in voltage clamp, or using a linear regression of voltage deflections in response to small current steps (between 5 pA and 50 pA), with equivalent results. Membrane time constants were determined by fitting an exponential curve to responses to the same small current steps at 0.2–500 ms from current onset. Action potential thresholds were defined as the voltage at which the slope trajectory reached 10 mV/ms (subtracting residual capacitive artifacts if necessary). Threshold values were averaged only for the smallest three superthreshold steps to minimize influence of uncompensated series resistance. Action potential amplitudes were measured from threshold. Afterhyperpolarization amplitudes were defined as the difference between action potential threshold and the minimum membrane potential attained during the afterhyperpolarization. Action potential parameters are given as the average over the first action potentials of a train. Saturating frequencies were determined as maximum spike number per current step; maximal frequencies were defined as the reciprocal of the minimum inter-spike interval. The amplitude of the sag current was determined for hyperpolarizing steps as the potential difference between the minimum and steady state voltages attained during current injection, and its time constant from a monoexponential fit between minimum and sag steady state decay. Both parameters depended linearly on the steady state voltage upon current injection, and values were interpolated to give the value at −100 mV to allow comparison. Irregular spiking was defined by the coefficient of variation (CV) of the inter spike intervals exceeding a threshold of 0.5. Delayed onset firing was defined by the delay of the first spike exceeding 100 ms. Strongly adapting cells were defined as cells that stopped firing before the end of the current injection. Parameters of firing accommodation are averages of the five longest spike trains. For early accommodation, the ratio of the first and the fifth spike parameter was taken; for late accommodation, it was the ratio between fifth and last spike. Statistical differences between interneuron groups were tested with an ANOVA, followed by a post-hoc Neuman-Keuls test. Differences were considered significant if p<0.05. Values are given as mean ± standard error.

### Morphology

After recordings, hippocampal slices were fixed in 4% paraformaldehyde in PBS overnight and multiple high resolution image stacks (typically 120×120 µm, 1024×1024 pixels; Δz = 0.75 µm) covering the entire cell, were taken with a Leica Confocal microscope (LCS SP2). Morphological reconstructions were produced manually from multiple image stacks. Morphological properties were quantified with ImageJ. Soma roundness was defined as 4π*area/perimeter^2^, with values between 0 and 1 and rounder somata closer to 1 [18]. The aspect ratio of the soma was defined as the ratio between the longest and shortest axis. Somata with aspect ratio >2 are sometimes referred to as fusiform [4], [18]. The dendritic tree was described by the number of principal dendrites, the verticality (ratio between radial and tangential dendritic span) and the orientation of its major axis with respect to the pyramidal cell layer (0° reflect a radial orientation similar to pyramidal cells, 90° is tangential). The axon was distinguished from the dendrites based on its thinner and smoother appearance. In some cases the axon was tortuous with ramifications and boutons could be discerned.

## Results

**Figure 1:**
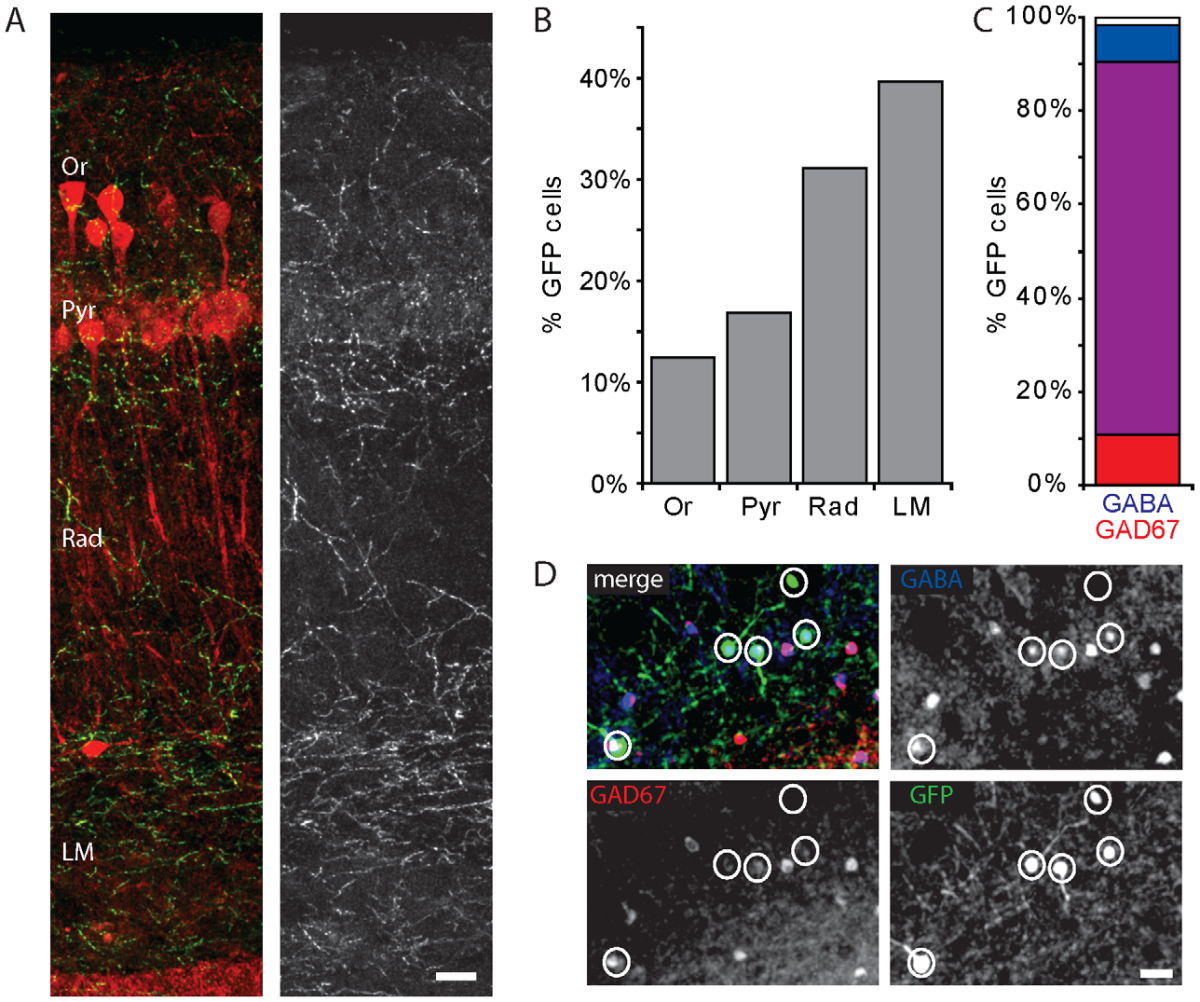
GFP-positive GABAergic interneurons. A: Maximal projection image illustrating the distribution of GFP-labeled profiles in the CA1 layers. Calbindin (red), labeling a fraction of pyramidal cells, is only shown to facilitate recognition of the layers. B: Distribution of GFP-labeled interneurons in the hippocampal CA1 area in GAD65-GFP mice. C: Percentage of GFP-labeled cells that expressed GABA (blue), GAD67 (red) or both (purple). Data from 268 GFP cells; 10 sections. D: Example of triple immunostaining for GFP (green), GABA (blue) and GAD67 (red). Scale bars are 30 µm. Abbreviations of CA1 layers: Or - oriens; Pyr – pyramidale; Rad – radiatum; LM – lacunosum moleculare.

This study was prompted by the observation that in acute hippocampal slices and slice cultures [19] of GAD65-GFP mice, the GFP-labeling was clearly non-uniform. We noticed denser labeling in the upper dendritic layers, stratum radiatum and stratum lacunosum-moleculare (Fig. 1A), suggesting that GFP-labeled interneurons in GAD65-GFP mice target preferentially dendrites of CA1 pyramidal cells. We therefore set out to characterize GFP-labeled interneurons in the hippocampal CA1 area in detail.

For a first characterization of GFP-labeled interneurons in GAD65-GFP mice, we performed immunohistochemistry for the inhibitory neurotransmitter GABA and the GABA-producing enzyme GAD67 in hippocampal sections of adult GAD65-GFP mice. GFP-positive neurons were present in all layers of the hippocampal CA1 area, with the highest density in the upper dendritic layers (Fig. 1B). Virtually all GFP-labeled cells were immuno-positive for GAD67 and/or GABA (Fig. 1C, D), confirming their identity as GABAergic interneurons [11]. This was also confirmed by the recordings of inhibitory currents in pairs of presynaptic GFP-labeled cells and postsynaptic CA1 pyramidal neurons (Müllner, Bonhoeffer and Wierenga, unpublished data).

### GFP-labeled interneurons contain reelin or VIP

**Figure 2:**
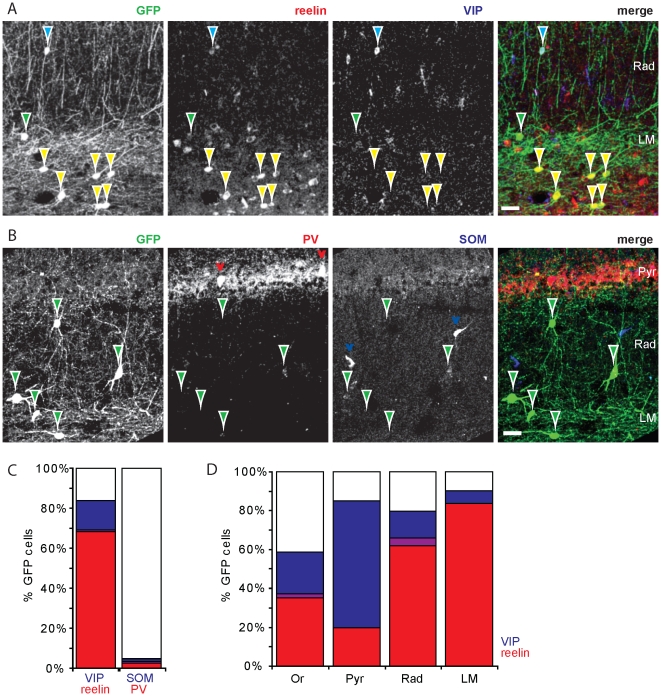
The majority of GFP-positive interneurons contain reelin or VIP. A: Immunohistochemistry for reelin (red) and vasoactive intestinal peptide (VIP; blue). Most GFP-positive interneurons (green) contained either reelin (yellow arrowheads) or VIP (blue arrowheads). Very few GFP-positive cells were lacking both (green arrowheads). B: Immunohistochemistry for parvalbumin (PV; red) and somatostatin (SOM; blue), showing minimal overlap with GFP-positive interneurons (green arrowheads). Red and blue arrowheads point to GFP-negative parvalbumin- and somatostatin-positive interneurons. C: Summary for all GFP-positive interneurons. D: Distribution of reelin- and VIP-containing GFP-labeled interneurons in the layers of the CA1 area. Overlap between both markers are indicated with purple. Scale bars are 30 µm.

Previous reports have suggested that the majority of GFP-labeled interneurons in GAD65-GFP mice originates from the caudal ganglionic eminence (CGE) [11], [20]. It was recently shown that most interneurons originating from the CGE express either reelin or vasoactive intestinal peptide (VIP) (Miyoshi et al., 2010), while interneurons that originate from the medial ganglionic eminence (MGE) are known to express either parvalbumin or somatostatin [8], [21]. To examine which interneurons express GFP in GAD65-GFP mice, we performed immunohistochemistry staining for reelin, VIP, parvalbumin and somatostatin in hippocampal sections. We found that 69±2% (n = 16 sections; 548 GFP cells) of GFP-positive interneurons in the CA1 area expressed reelin (Fig. 2A, C). The percentage of GFP cells that expressed reelin differed between layers (Fig. 2D). In particular, >80% of GFP-expressing cells in stratum lacunosum-moleculare showed strong reelin expression. Furthermore, 15±2% of GFP-positive interneurons expressed VIP (n = 9 sections; 313 cells; Fig. 2A, C), most of which were located close to or in the pyramidal layer (Fig. 2D). In agreement with the previous report by Miyoshi and coworkers [6], reelin and VIP expression did not overlap (1±1%; n = 9 slices; 313 GFP cells). Virtually no GFP cells were found that expressed parvalbumin (2±1%; n = 14 slices; 437 GFP cells) or somatostatin (3±1%; n = 27 slices; 727 GFP cells), markers that are typical for interneurons derived from the MGE (Fig. 2B, C). The high occurrence of reelin and (to a lesser extent) VIP indicates that the vast majority of GFP-labeled interneurons in GAD65-GFP mice originates from the CGE.

The fraction of GFP-interneurons expressing these four cellular markers only accounts for up to 90% of the total population. The remaining 10% might simply reflect unavoidable imperfections of immunohistochemistry experiments and analysis. Alternatively, these cells could reflect a separate class of CGE-derived interneurons for which a marker is presently lacking [6].

### Molecular profiles

We next wanted to examine the molecular profile of GFP-positive cells in the hippocampal CA1 area in more detail. We examined the expression of the classical interneuron markers neuropeptide Y (NPY), CCK, calretinin and calbindin. We found, in agreement with earlier reports on CGE-derived interneurons, that GFP-labeled cells expressed a variety of interneuron markers.

**Figure 3:**
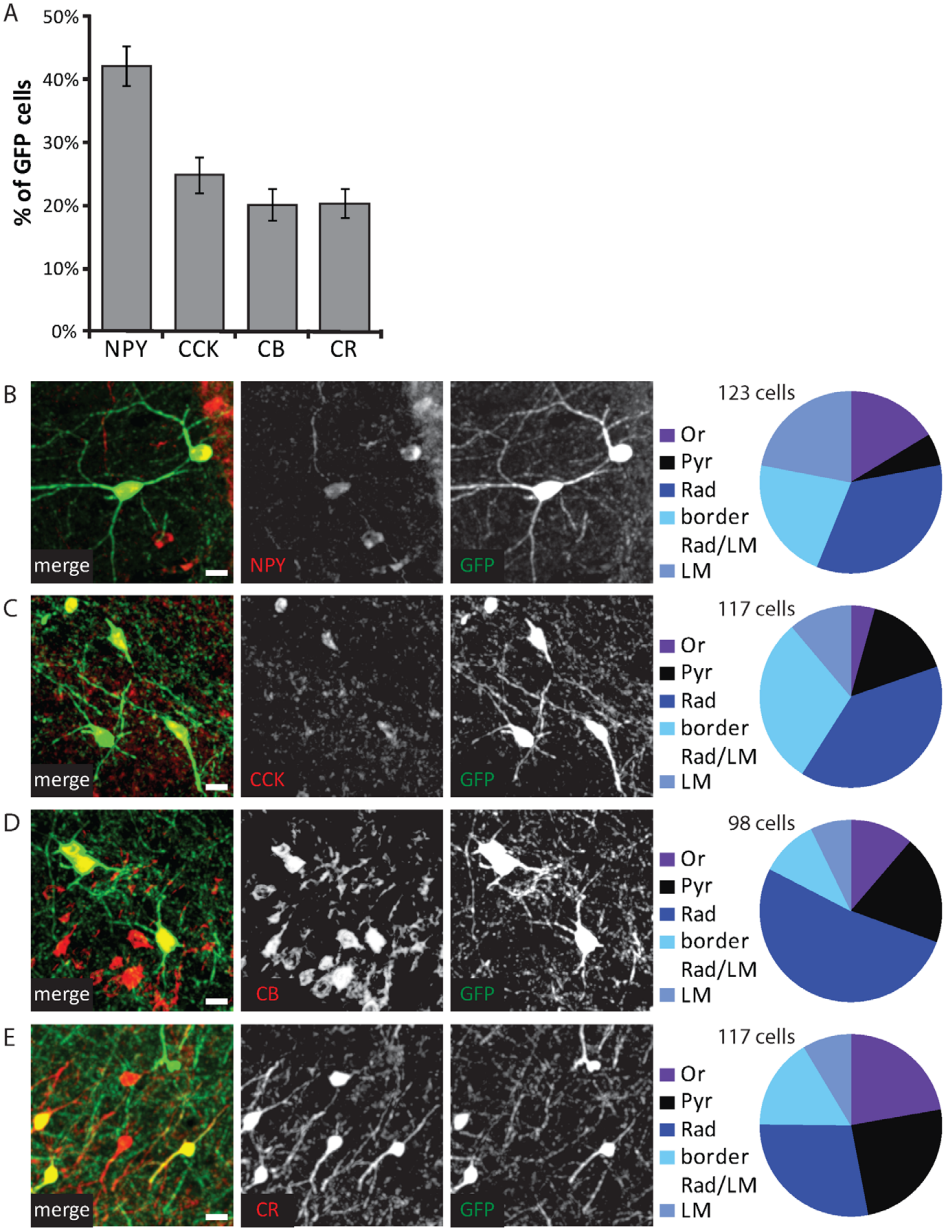
Molecular profile of GFP-positive interneurons. A: Percentage of GFP-labeled interneurons that contained neuropeptide Y (NPY), cholecystokinin (CCK), calbindin (CB) and calretinin (CR). Data from 18–21 sections and 350–550 GFP cells were examined for each label; error bars represent standard error. B–D: Left: Examples of GFP-labeled interneurons, containing NPY (B), CCK (C), calbindin (D; CB) or calretinin (E; CR). Right: Distribution of positive GFP cells over the CA1 layers. Scale bars are 10 µm.

The largest subgroup consisted of GFP cells that expressed NPY. 42±3% of GFP cells expressed NPY (Fig. 3A; n = 18 sections; 368 GFP cells), and 22±2% of NPY-positive interneurons were labeled with GFP. Although almost 30% of all NPY-positive interneurons co-expressed somatostatin (166/595), the subset of GFP-expressing cells did not (304/310; 98%). NPY-expressing GFP cells were present in all layers of the hippocampal CA1 area and they had mostly multipolar morphologies (73/123; 59%; Fig. 3B). NPY-expressing GFP cells (31/123; 25%) with tangentially oriented morphologies were mostly located in the upper dendritic layers stratum lacunosum-moleculare and stratum radiatum. The labeled cell types correspond well to previously described NPY-positive multipolar cells, often referred to as Ivy cells and neurogliaform cells [20], [22], [23]. NPY-positive CA1 interneurons mainly target the shafts of pyramidal cell dendrites [22], [24].

In a few sections, we performed co-staining of NPY and reelin. We found that reelin expression was slightly higher in NPY-positive GFP cells compared to the general population of NPY-positive interneurons (75% vs. 64% reelin-positive cells; p<0.05, χ^2^-test; n = 3 sections). One possible explanation for this finding is that different populations of NPY-expressing cells exist [20] and that those populations are not equally represented in the GFP-labeled interneurons.

CCK was expressed in 25±3% of GFP cells (Fig. 3A) and GFP-labeled interneurons accounted for 35±3% of CCK-expressing cells in the hippocampal CA1 area (n = 20 sections; 518 GFP cells). The vast majority of CCK-positive GFP cells were located in the dendritic regions stratum radiatum and stratum lacunosum-moleculare (94/117; 80%; Fig. 3C). CCK-positive GFP cells in stratum radiatum had mostly multipolar morphologies (31/46; 67%) and ∼50% of them co-expressed calbindin (24/46; 52%). In the upper dendritic layers (at the border between stratum radiatum and stratum lacunosum-moleculare and within stratum lacunosum-moleculare), dendrites and somata of CCK-positive GFP cells were oriented tangentially (24/48; 50%) or multipolar (17/48; 35%). These interneurons correspond well with previously described CCK-positive interneuron types that are known to innervate CA1 pyramidal dendrites [25], [26]. They are often divided according to their main targeting area and referred to as Schaffer collateral-associated, perforant path-associated and apical dendrite innervating cells [27]. In addition, a small group of CCK-positive but calbindin-negative GFP cells (12/102; 12%) with mostly radially oriented morphology was located close to the pyramidal cell layer. This last group fits the description of CCK-positive basket cells [13], [28], which are known to target pyramidal cell somata and dendrites [25].

Expression of calbindin was found in 20±2% of GFP cells (Fig. 3A; n = 20 sections; 518 GFP cells). Calbindin was also expressed by a subset of CA1 pyramidal neurons, which made it impossible to unambiguously distinguish between calbindin-positive GFP-negative interneurons and principal cells. We therefore could not determine what fraction of calbindin-positive interneurons were labeled by GFP in our sections. Calbindin-expressing GFP cells were mostly located around the pyramidal layer and in stratum radiatum (70/98; 71%) and had multipolar morphologies (Fig. 3D). Calbindin-expressing interneurons have been shown to specifically target the dendrites of pyramidal cells [29]. A few (5/84; 6%) large, tangentially oriented GFP cells in stratum oriens were also calbindin-positive. These interneurons have been described to have long-range projections to the septum [29], [30]. They also make local projections to pyramidal cell dendrites [31], [32] and possibly interneurons [33].

Of all CCK-expressing cells, 40±4% co-expressed calbindin. This percentage was similar in CCK-expressing GFP cells (37%; p = 0.27, χ^2^-test; n = 20 sections; 518 GFP cells), suggesting no specific preference. GFP cells co-expressing calbindin and CCK were located in stratum radiatum and had mostly multipolar or radially oriented dendrites.

Finally, 20±2% of GFP cells expressed calretinin (Fig. 3A), and 11±1% of all calretinin-expressing cells were GFP-labeled (n = 21 sections; 557 GFP cells). Calretinin-expressing GFP cells were located mostly in or close to the pyramidal layer (Fig. 3E). Half of the calretinin-expressing GFP cells (46/92; 50%) had radially oriented, bipolar morphologies. These interneurons have been described before and are known to often co-express VIP and specifically target dendrites of other interneurons in stratum radiatum and stratum oriens [22], [34], [35]. The other calretinin-containing GFP cells had mostly multipolar morphologies (38/92; 41%). Although in the neocortex a subset of calretinin-expressing interneurons is known to target dendrites of principal neurons [36], [37], it is not known whether such interneurons also exist in the hippocampus.

The few GFP cells that expressed parvalbumin were located around the pyramidal layer (8/8; 100%) and had typical radial or multipolar morphologies, as has been described before for parvalbumin-positive basket cells [2], [38]. The somatostatin-expressing GFP cells were concentrated in stratum oriens and showed mostly tangential morphologies (11/16; 69%), also in agreement with previous reports [2], [38].

These immunohistochemistry results indicate that GFP-labeled interneurons in GAD65-GFP mice display a variety of classical interneuron markers and morphologies. However, despite this variation the emerging common theme is that the vast majority of GFP-labeled interneurons (the exception being soma-targeting CCK-positive basket cells and interneuron-targeting calretinin-positive bipolar cells) innervate the dendrites of CA1 pyramidal neurons.

### Firing properties

**Figure 4:**
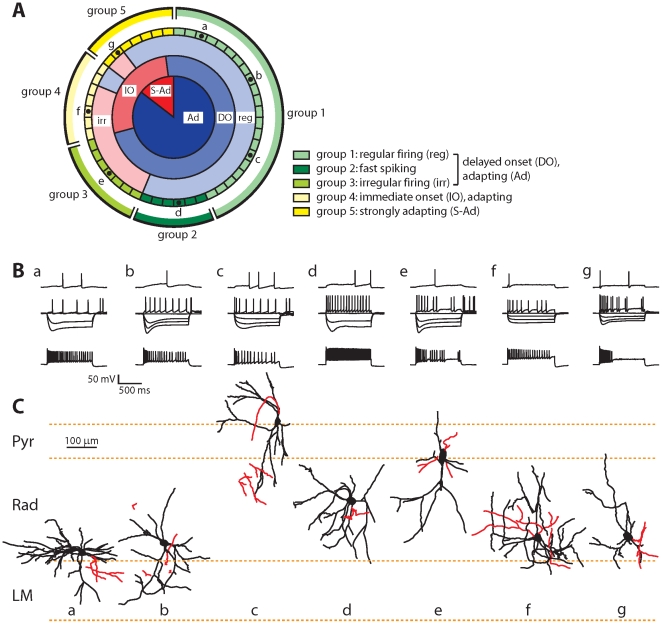
Firing properties of GFP-positive interneurons. A: Representation of all recorded GFP-labeled interneurons, arranged according to characteristics of their firing patterns and their classification in 5 groups. Each segment represents a single interneuron. Inner ring: adapting (Ad; dark blue) and strongly adapting (S-Ad; dark red) cells. Second ring: cells showing delayed onset (DO; blue) and immediate onset (IO; red) firing. Third ring: cells displaying regular (reg; light blue) and irregular (irr; light red) firing. Interneurons were divided in five groups (1–5) as indicated with yellow and green colors. The letters correspond to example cells (a–g) in B and C. B: Examples of firing patterns of example cells a–g (as indicated in A). Upper traces show firing around threshold, middle traces show responses to hyperpolarizing steps (100 pA step size) and intermediate firing and lower traces show maximal firing. C: Morphology of example cells a–g (same as in B). Dendrites are shown in black, axons in red.

To further characterize the GFP-labeled interneurons, we recorded firing patterns in whole-cell patch clamp mode in acute slices of GAD65-GFP mice. The firing patterns of the majority of GFP-labeled interneurons were characterized by delayed onset firing around threshold and continuous, regular firing at higher firing frequencies with moderate frequency adaptation (‘adapting’). However, these three features were not perfectly overlapping and mixed firing patterns were also found (Fig. 4A, B).

We grouped the recorded cells (n = 48) in five subclasses according to their firing patterns. Delayed onset, adapting interneurons (n = 34) were subdivided into three subclasses: regular firing interneurons (21/34; 62%; group 1 in Fig. 4A, Ba–c), fast spiking interneurons (6/34; 18%; group 2 in Fig. 4A, Bd), and irregular spiking interneurons (7/34; 21%; group 3 in Fig. 4A, Be). Fast spiking interneurons were defined as having firing rates >50 Hz. They had shorter action potential widths and frequently displayed rebound action potentials after hyperpolarizing current injections (Table 1). Irregular spiking interneurons were defined by their large variance in inter-spike intervals (coefficient of variation >0.5). They tended to have slightly more hyperpolarized resting membrane potentials, but were otherwise very similar to group 1 interneurons (Table 1). In most delayed onset cells (27/34; 79%), the delayed firing remained when the cell fired 2 or 3 action potentials (corresponding to the LS1 type of Miyoshi *et al.* [6]). The fourth group of interneurons did not exhibit delayed onset firing at threshold and displayed mostly irregular firing patterns (7/48; 15%; group 4 in Fig. 4A, Bf). They distinguished themselves by a fast membrane time constant, low input resistance and low action potential threshold (Table 1). Finally, the fifth group consisted of strongly adapting interneurons (7/48; 15%; group 5 in Fig. 4A, Bg), which stopped firing prematurely before the end of the current injection. They had small action potential amplitudes and fast sag current kinetics (Table 1). None of the recorded GFP-labeled interneurons showed intrinsic burst firing.

**Table 1:** Electrophysiological properties of GFP-labeled interneurons.

	Delayed onset, adapting				
	Regular spiking(n = 12)	Fast spiking(n = 5)	Irregular spiking(n = 4)	Immediate onset, adapting(n = 6)	Strongly adapting(n = 5)
	group 1	group 2	group 3	group 4	group 5
Resting membrane potential (mV)	−49±1	−51±2	−55±2	−58±2	−51±2
	*group 4< group 1,2,5 (p = 0.002)*				
Input resistance (MΩ)	468±30	377±54	401±50	253±50	372±59
	*group 4< group 1 (p = 0.014)*				
Membrane time constant (ms)	63±7	36±13	57±12	21±12	34±14
	*group 4< group 1 (p = 0.028)*				
Action potential threshold (mV)	−35±1	−35±2	−35±2	−42±2	−35±2
	*group 4< group 1–3,5 (p = 0.007)*				
Action potential amplitude (mV)	78±2	75±4	−76±4	−80±4	65±4
	*group 5< group 1,2,4 (p = 0.041)*				
Action potential halfwidth (ms)	1.6±0.1	1.2±0.1	1.7±0.1	1.2±0.1	1.6±0.1
	*group 2< group 1 (p = 0.028)*				
Afterhyperpolarization (mV)	−19±1	−22±2	−17±2	−16±2	−19±2
	
CV of inter spike interval	0.3±0.05	0.2±0.1	0.6±0.1	0.7±0.1	0.4±0.1
	*group 3,4> group 1,2,5 (p<0.001)*				
Saturating Frequency (Hz)	30±2	59±4	19±3	21±3	18(4
	*group 2> group 1,3–5; group 1>3,5 (p<0.001)*				
Maximal Frequency (Hz)	79±5	120±9	87±9	89±9	66±9
	*group 2> group 1,3–5 (p = 0.002)*				
Delay to first spike (ms)	496±40	525±80	513±68	29±73	70±78
	*group 4< group 1–3; group 5< group 1–3 (p<0.001)*				
Rebound spikes (% of cells)	57±10	83±19	14±18	14±18	67±19
	*group 2> group 3,4 (p = 0.023)*				
Sag amplitude at −100 mV (pA)	17±2	19±4	13±4	10±4	22±4
	
Sag decay time constant at −100 mV (ms)	225±20	158±35	238±39	271±32	104±39
	*group 5< group 1,3,4 (p = 0.017)*				
Early frequency accommodation (%)	55±4	67±8	52±7	59±7	65±8
	
Late frequency accommodation (%)	66±4	71±7	62±6	82±6	84±7
	
Early amplitude accommodation (%)	73±3	79±5	71±5	77±5	70±5
	
Late amplitude accommodation (%)	84±3	82±5	86±4	81±4	83±5
	
Early AP halfwidth accommodation (%)	166±7	143±14	195±13	126±13	157±14
	*group 3> group 2,4 (p = 0.006)*				
Late AP halfwidth accommodation (%)	116±3	125±5	117±4	121±4	109±5

Statistical differences with between groups were tested with ANOVA, followed by a post-hoc Neuman-Keuls test. Significant differences with corresponding p-values (ANOVA) are indicated in red. Values are given as mean (standard error. Abbreviations: CV – coefficient of variation, AP – action potential.

### Interneuron morphology

We also quantified morphological parameters of the recorded GFP cells, but we were unable to detect a strict correlation between electrophysiological and morphological properties (Fig. 4C and Table 2). However, 4 of 6 cells that had morphologies similar to those described for CCK-positive basket cells (i.e. soma location close to pyramidal layer, axon targeting pyramidal layer, radial orientation; e.g. Fig. 4Ce) showed irregular firing [39]. The exceptions were a strongly adapting and a regular firing interneuron. In addition, 10 of 11 interneurons with morphologies similar to neurogliaform cells (soma location in deep dendritic layers, compact multipolar morphology, axon ramifying close to soma; Fig. e.g. 4Ca,b) displayed delayed onset firing. The one exception was a strongly adapting interneuron.

**Table:** **Table 2.** Morphological properties of GFP-labeled interneurons.

	Delayed onset, adapting				
	Regular spiking(n = 12)	Fast spiking(n = 5)	Irregular spiking(n = 4)	Immediate onset, adapting(n = 6)	Strongly adapting(n = 5)
	group 1	group 2	group 3	group 4	group 5
Roundness soma	0.6±0.2	0.8±0.04	0.6±0.1	0.6±0.1	0.7±0.2
Aspect ratio soma	2.2±0.8	1.5±0.3	2.1±1.1	2.0±0.7	1.6±0.3
Verticality of dendrites	0.8±0.4	1.1±0.3	1.1±0.5	1.4±0.6	0.8±0.3
Orientation (degrees)*	59±31	61±27	37±37	24±25	71±19
Number of primary dendrites	4.1±1.2	4.2±1.6	5.5±2.6	4.4±0.9	4.2±0.4

*Tangential cells have an orientation close to 90 degrees; 0 degrees reflect radially oriented cells (similar to pyramidal cells).

No significant differences in morphological parameters between groups were detected (ANOVA). Values are given as mean ± standard error.

Although we were not able to reconstruct the entire axonal arbors, in most interneurons we could detect some axonal ramifications and/or boutons, indicative of the axonal projection area. In agreement with our immunostaining findings, the majority of GFP-labeled interneurons (24/32; 75%) had axons ramifying in the dendritic layers (stratum radiatum and stratum lacunosum-moleculare).

## Discussion

We characterized GFP-labeled interneurons in the hippocampal CA1 area of GAD65-GFP mice. We found that the majority of labeled interneurons most likely have their origin in the CGE. While different subtypes of interneurons are labeled in this mouse line, we found a strong bias towards reelin-expressing, dendritically targeting interneurons with firing patterns showing delayed onset firing around threshold and moderate firing adaptation at higher firing frequencies.

The CGE was found to generate ∼30–40% of interneurons in the somatosensory cortex and reelin- and VIP-expressing interneurons in a ∼1∶1 ratio [6], [7]. In the present study, we found that GFP-labeled interneurons in the GAD65-GFP mice form only ∼20% of CA1 interneurons and showed a strong bias towards reelin-expressing interneurons (ratio reelin:VIP was ∼4∶1–5∶1). This could reflect regional differences between hippocampus and neocortex, but could also indicate that in the GAD65-GFP mice GFP is expressed in only a subset of CGE-derived interneurons with a bias towards reelin-expressing interneurons. This is further supported by our electrophysiological recordings, where we only find a subset of firing pattern classes previously described for CGE-derived interneurons [6]. The vast majority of the recorded cells showed a slowly adapting firing pattern with delayed firing around threshold. Indeed, such firing patterns were previously described as typical for reelin-expressing CGE-derived interneurons [6]. Intrinsic bursting interneurons, which were reported to represent >20% of CGE-derived cells, were not found in our study.

We found that less than 5% of GFP-labeled interneurons in GAD65-GFP mice expressed parvalbumin or somatostatin. These cells were brightly labeled and their location and morphologies were consistent with the well-described characteristics of parvalbumin- and somatostatin-positive interneurons [2], [38]. It is therefore unlikely that these were false positives of the immunohistochemistry. Labeling of a few parvalbumin and somatostatin interneurons could indicate that a small minority of MGE-derived interneurons also express GFP in the GAD65-GFP mice. It was recently shown that the preoptic area also contributes to the generation of cortical and hippocampal interneurons [10], albeit for only 5–10%. Preoptic area derived interneurons express mostly reelin (but not VIP), but also some parvalbumin- and somatostatin-positive interneurons were reported to origin from the preoptic area [9]. Therefore, an alternative explanation for our findings would be that interneurons from the preoptic area also express GFP in GAD65-GFP mice. This would also contribute to the bias towards labeling of reelin-expressing interneurons. Specific markers for preoptic area derived interneurons will be necessary to distinguish between these possibilities in the future.

The majority of the GFP-labeled interneurons were located around the border of stratum radiatum and lacunosum-moleculare. It has been reported previously that interneurons in that location mainly project to pyramidal cell dendrites [26]. Based on our immunohistochemical and morphological findings, we conclude that GFP-labeled interneurons include several previously described subtypes, which are known to mainly project to dendrites [27]. The largest class consists of NPY-positive Ivy and neurogliaform cells [20], [22]. GFP-labeled interneurons further include CCK-positive interneurons targeting the somata (i.e. basket cells) or dendrites (e.g. Schaffer-associated, perforant path-associated cells) of CA1 pyramidal cells [25], [40], [41], and calbindin-positive cells [29]. Despite the fact that the anatomy and immunohistochemistry of these dendritically-targeting interneurons have been described in detail, their precise function is generally more elusive [27]. In particular, it is not known if interneurons expressing different chemical markers execute different functions in the CA1 circuitry. The finding that most GFP-labeled interneurons target pyramidal dendrites makes GAD65-GFP mice well suited for studies focused on dendritic inhibition. At the same time, these mice also allow selection of specific interneuron subtypes based on known morphology and/or firing properties (e.g. CCK-positive basket cells [13], [28] or reelin-positive neurogliaform cells at the radiatum/lacunosum-moleculare border).

The main exception to the dendrite-targeting rule is formed by GFP-labeled calretinin-expressing interneurons, which have been shown to preferentially target other CA1 interneurons and avoid pyramidal cells [35]. In the neocortex, a subset of calretinin-positive interneurons preferentially innervates principal cells [36], [37], but such cells have not been described for the hippocampus. It has been suggested for some interneurons that they only transiently innervate interneurons during postnatal development, before switching to dendrites of pyramidal cells in the adult brain [32]. It would be interesting to examine whether something similar occurs in (a subset of) calretinin-expressing interneurons.

The majority of GFP-labeled interneurons in GAD65-GFP mice, especially those in the dendritic layers, express reelin. Reelin is essential for the proper layering of principal cells during development, but the function of reelin in the adult brain is more enigmatic [42], [43]. Reelin function in the adult brain has been linked to cytoskeleton stabilization [44], [45], as well as maintenance of NMDA receptors [16] and spines [46], [47]. Interestingly, the description of GFP-labeled interneurons in GAD65-GFP mice has a remarkable overlap with interneurons that express the serotonin receptor 5HT3 [48]. Reelin and 5HT3 receptors are components of a common pathway controlling the postnatal development of apical dendrites of neocortical principal cells [49]. The GAD65-GFP mice could be a useful tool to investigate the role of reelin produced by adult interneurons.
